# Pharmacogenetics of Biological Agents Used in Inflammatory Bowel Disease: A Systematic Review

**DOI:** 10.3390/biomedicines9121748

**Published:** 2021-11-23

**Authors:** Rita Lauro, Federica Mannino, Natasha Irrera, Francesco Squadrito, Domenica Altavilla, Giovanni Squadrito, Giovanni Pallio, Alessandra Bitto

**Affiliations:** 1 Department of Clinical and Experimental Medicine, University of Messina, Via C. Valeria, 98125 Messina, Italy; rita.lauro@studenti.unime.it (R.L.); fmannino@unime.it (F.M.); nirrera@unime.it (N.I.); fsquadrito@unime.it (F.S.); giovanni.squadrito@unime.it (G.S.); abitto@unime.it (A.B.); 2 SunNutraPharma, Academic Spin-Off Company of the University of Messina, Via C. Valeria, 98125 Messina, Italy; daltavilla@unime.it; 3 Department of Biomedical, Dental, Morphological and Functional Imaging Sciences, University of Messina, Via C. Valeria, 98125 Messina, Italy

**Keywords:** Inflammatory Bowel Disease, Crohn’s disease, ulcerative colitis, infliximab, adalimumab, vedolizumab, ustekinumab, polymorphism

## Abstract

Inflammatory Bowel Disease (IBD) comprises a group of disorders, in particular Crohn’s disease (CD) and ulcerative colitis (UC), characterized by chronic inflammation affecting the gastrointestinal tract. The treatment of these conditions is primarily based on anti-inflammatory drugs, although the use of biological drugs with lower side effects quickly increased in the last decade. However, the presence of certain polymorphisms in the population may determine a different outcome in response to therapy, reflecting the heterogeneity of the efficacy in patients. Considering that several studies showed important correlations between genetic polymorphisms and response to biological treatments in IBD patients, this systematic review aims to summarize the pharmacogenetics of biologicals approved for IBD, thus highlighting a possible association between some polymorphisms and drug response. With this purpose, we reviewed PubMed papers published over the past 21 years (2000–2021), using as the search term “drug name and IBD or CD or UC and polymorphisms” to underline the role of pharmacogenetic tests in approaching the disease with a targeted therapy.

## 1. Introduction

Inflammatory Bowel Diseases (IBDs) are a group of inflammatory disorders of the gastrointestinal tract, with Crohn’s disease (CD) and ulcerative colitis (UC) being the most widely represented types. IBD can begin at any age and regardless of sex, but the most important peak of onset is concentrated between 15 and 45 years, although a second peak can be found at a later, elderly age. These are severely invalidating conditions, and the incidence is progressively increasing, enough to be considered a global healthcare problem [1,2,3].

IBDs are diseases of unknown cause arising spontaneously, although an aberrant dysregulation of the immune response toward commensal and nonpathogenic antigens normally found in the gut is the prevailing pathogenic hypothesis [4]. This might be ascribed to the individual genetic susceptibility and/or to microbiomic and environmental factors. An altered interaction between the host and the microbes results in an immunological imbalance which promotes the production of autoreacting cell clones [4,5]. Particularly, the activation of molecular pathways such as Nuclear Factor Kappa-B (NF-κB), Mitogen Activated Protein Kinase (MAPK), and Tumor Necrosis Factor-α (TNF-α) causes activation of effector T cells and massive production of pro-inflammatory molecules as Interleukin 12 (IL-12) and IL-23, and interferon γ (IFN-γ). Consequently, the continuous activation of the macrophage-T lymphocyte axis in an autocrine manner, results in lymphocyte accumulation, increased interaction between endothelial adhesion molecules and integrins, and epithelial disruption [6,7,8]. This epithelial damage stimulates fibroblast release of metalloproteases that by degrading the connective tissue, promote ulcer formation [9].

Up to date, there is no definitive therapy for IBDs and, generally, the severity of the disease drives towards the most appropriate therapeutic approach. The main treatment options aim to reduce intestinal inflammation, to relieve symptoms, to keep the disease in remission and prevent its acute exacerbations. Indeed, pharmacological therapy for these disorders involves aminosalicylates, corticosteroids, and immunosuppressants [10]. However, although knowledge of the pathophysiology of IBDs has not been fully elucidated, significant advances have allowed the development of new and more promising therapies with lower side effects. For example, the development of biological agents allowed to target single steps in the immune cascade, to modulate the underlying inflammatory mechanism.

Targeting TNF-α reflects its importance in mediating the T-lymphocyte-driven immune response, and the use of monoclonal antibodies to neutralize TNF-α interfere with the inflammatory response and decrease the frequency of flare-ups in two-thirds of patients with moderate and/or severe CD [11] and UC [12]. Anti-TNF therapies that have been used in the clinical setting of IBD include infliximab, adalimumab and golimumab [13], all with distinct pharmacodynamic profiles and variable efficacy [14,15]. These agents improved both remission and maintenance, especially for patients with CD. However, approximately one-third of IBD patients are either nonresponsive or lost treatment efficacy over time, and genetic factors are responsible for this inability. Improvements in genetic characterization techniques and genome-wide association studies (GWASs) allowed the identification of genetic variants which could influence the development of the disease, the response to treatment, and the development of adverse effects [16].

Several studies showed that some single nucleotide polymorphisms (SNPs) in the gene encoding TNF-α itself can influence the anti-TNF response in patients with IBD. TNF-α is known to be associated with an increased risk of developing IBD. Therefore, several studies demonstrated an association between polymorphisms and different pharmacological responses to treatments, although some results appear to be controversial [17,18,19]. Same studies investigated the role of TNF Receptor 1 (TNFR1) and 2 (TNFR2) in the response to anti-TNF. These proteins are encoded from TNF-α receptor superfamily 1A (TNFRSF1A) and TNF-α receptor superfamily 1B (TNFRSF1B) genes, and mediate pro-apoptotic and pro-inflammatory responses when bound by TNF-α, thus regulating the correct functioning of the immune system [20]. Indeed, it is known that mutations in these genes are related to the development of some autoimmune diseases, including CD and UC [8,21].

Genes encoding proteins that are implicated in the immune response have been a further research target to evaluate responses to anti-TNF agents. For example, Nucleotide-Binding Oligomerization Domain containing protein-2 (NOD2), also known as Caspase Recruitment Domain-containing protein 15 (CARD15) gene, is able to control the innate immune system by functioning as a Pathogen Recognizing Receptor (PRR), subsequently influencing TNF-α expression [22,23,24]. Therefore, its variants could regulate the response to anti-TNF-α therapies. Another protein-coding gene which plays an important role in the recognition of pathogens molecular patterns is Toll Like Receptor 4 (TLR4), renowned for its implication in the pathogenesis of IBD [25]; recent findings have also highlighted a role for the CD14 gene for its protective effect of IBD, because it acts as an orchestrator of the intestinal mucosal barrier homeostasis [26]. Moreover, polymorphisms affecting the levels of pro-and anti-inflammatory cytokines can influence the pathophysiology of IBD. For this reason, various mutations upon cytokines genes, especially IL-1β, IL-1RA, IL-6, IL-11, IL-13, IL-17, and IL-27 have been investigated.

Additional emphasis was laid on genes involved in apoptosis and autophagy for their involvement in response to TNF-inhibitors, which affects the inflammatory immune response in IBD [27]. Among these genes, Fas and Fas-ligand (FasL) are particularly important in the downregulation of immunological processes [28]. As a matter of fact, an increased expression of FasL in the lamina propria lymphocytes contributes to the mucosal injury in UC. Another gene whose variants may affect treatment response is Caspase-9 (CASP9) encoding for the homonymous protein that is an initiator of apoptosis. Furthermore, some mutations of Autophagy-Related 16-like 1 (ATG16L1) gene, an essential component of the autophagic pathways [29], have been reported to relate with the development of IBD, particularly CD [30]. For all the mentioned reasons, variants in these latter described genes have been studied for any implication with clinical responses to anti-TNF agents.

Other newly approved targets to treat IBD are the p40 subunit of IL-12 and IL-23. Ustekinumab is a monoclonal antibody against IL-12 and IL-23 approved for the treatment of several autoimmune disorders, including CD, which induced response already after 8 weeks and maintained clinical benefits up to 52 weeks after treatment [31,32]. Ultimately, a new therapeutic agent used to treat IBDs aims to block the action of integrins to inhibit leukocyte trafficking at the inflammatory site, thus reducing their activities and preventing the elevated inflammatory response. Therefore, vedolizumab, a fully humanized monoclonal antibody against α_4_β_7_ integrin, was approved for the treatment of adult patients with moderate-to-severe active CD and UC [33,34].

To understand the role of pharmacogenetics in the treatment of IBDs we reviewed the current literature in order to provide a basis for a proper use of biological therapies in IBD patients.

## 2. Methodology

### 2.1. Search Strategy

Studies were identified, screened and extracted for relevant data following the PRISMA (Preferred Reporting Items for Systematic Reviews and Meta-Analyses) guidelines. Literature search, title, abstract and full text screening were conducted independently by RL and GP. PubMed was used to retrieve articles published in the last 21 years (2000–2021) and search terms were “infliximab and polymorphism and Crohn’s disease”, “infliximab and polymorphism and ulcerative colitis”, “adalimumab and polymorphism and Crohn’s disease”, “adalimumab and polymorphism and ulcerative colitis”, “ustekinumab and polymorphism and Crohn’s disease”, “ustekinumab and polymorphism and ulcerative colitis”, “vedolizumab and polymorphism and Crohn’s disease”, “vedolizumab and polymorphism and ulcerative colitis”, “golimumab and polymorphism and ulcerative colitis”.

### 2.2. Inclusion and Exclusion Criteria

Papers that fulfilled the following inclusion criteria were included: articles on adults or pediatric patients of any gender and ethnicity receiving one of the biological agents used in IBD treatment independently from difference efficacy in drug response. Irrelevant studies were excluded if: (i) biological agents were used to treat other diseases, (ii) articles were written in a language other than English, (iii) papers with unspecified genotypes.

### 2.3. Extraction of Relevant Data, Quality and Risk of Bias Assesment

Relevant data was extracted and compared through a data extraction sheet. Extraction procedure was conducted by DA and GS. Extracted data included (i) disease, (ii) number of patients, (iii) polymorphic locus, (iv) biological agent, (v) clinical effects, (vi) duration of the study. Missing data entries were marked with N/A (not applicable).

The quality of the studies was assessed using the Newcastle–Ottawa Score for the observational studies [35] and the Cochrane Risk of Bias tool for the RCTs [36].

## 3. Results

### 3.1. Systematic Review

**Figure 1:**
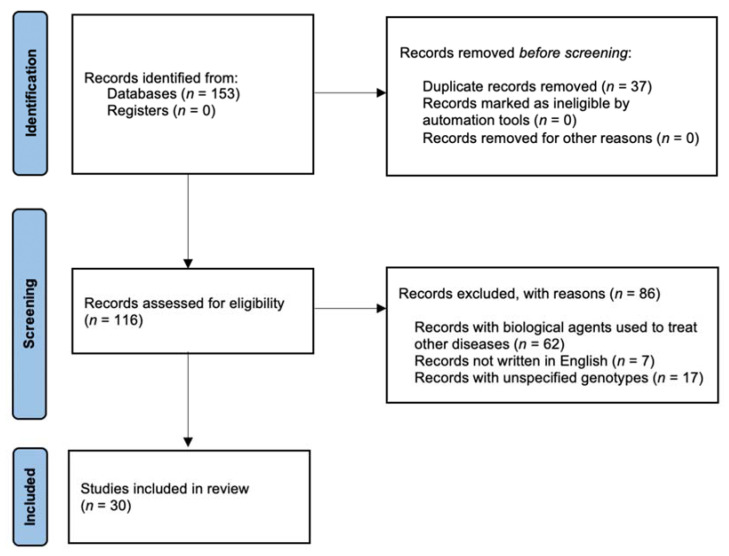
Stratification of papers considered in this review.

A number of 153 results were retrieved from Pubmed, and 116 papers were included in title and abstract screening after duplicates were removed. No records were marked as ineligible by automation tools nor other records were removed for other reasons in this phase. Overall, 116 articles were screened, and a total of 30 studies were included for data extraction (Figure 1). The included studies represented a wide range of polymorphisms that could influence biological treatment in IBD, such as in TNF-α and TNFR1/2 genes (n = 15), in innate immunity related genes (n = 9), in apoptosis and autophagy genes (n = 4), and in PTPN2 gene (n = 2).

### 3.2. Polymorphisms of TNF-α and TNFR1/2 Genes

Promoter-level polymorphisms in TNF-α gene, often associated with increased cytokine secretion, would appear to be a plausible explanation for the inefficiency of anti-TNF treatment [37,38]. In particular, TNF -308 (rs1800629) polymorphism has been associated with a modulated secretion of the cytokine, where a minor allele (A) is considered a potent transcriptional activator which enhances TNF-α production, compared with a more common allele (G), therefore promoting a worse clinical response to infliximab or adalimumab [38]. Balog et al., instead, conducted a study in 14 patients affected by chronic active CD and unresponsive to infliximab therapy, confirming the role of the A allele in rs1800629 polymorphism in the induction of a non-responder profile [39]. Similar consequences have also been reported in a prospective cohort study with 121 patients recruited, with 21 of them being non-responders to infliximab treatment: the presence of the A allele in TNF-α -308 gene was associated with three-fold higher odds of being a non-responder (*p* = 0.049) [40]. Moreover, in 82 Spanish CD and UC patients, an increased frequency of the A allele was found within non-responder patients (*p* < 0.05). This would highlight the role of the A nucleotide as responsible for the clinical ineffectiveness of anti-TNF-α therapy [41], but in another study on 236 CD patients from Belgium no significant difference has been highlighted between responders and non-responders regardless of -308 polymorphisms [42].

Another TNF-α variant, in position -238, rs361525, has been profoundly studied for its incidence in response to anti-TNF agents, in a meta-analysis comprehensive of 532 studies. In this case, the presence of the G allele, considered a common allele, has been associated with a positive response to these agents in the overall population (*p* = 0.011) and in Caucasians (*p* = 0.016). Moreover, in the same study, a positive correlation between the TNF-α -308 G allele and response to TNF-α inhibitors was found (*p* = 0.0001) [43].

Furthermore, the overall assessment between a common C allele versus a minor T allele in the TNF-α -857 polymorphism, rs1799724, was also estimated. Patients with the -857 C common allele showed a better response to TNF-α inhibitors than those having the minor allele (*p* = 0.003) [43]. In a total of 121 Japanese CD patients, a decrease in response to infliximab therapy in patients with the minor (T) allele of TNF-α -857 compared with the more common (C) allele has been demonstrated [44]. However, another study found no association between the presence of all the previous polymorphisms and clinical response to anti-TNF-α agents in CD and UC patients [45]. Same conclusions were obtained studying -238 G/A, -308 G/A, and -857 C/T polymorphism in the promoter region of TNF-α in a Greek cohort of 79 adults and 27 children with CD [46].

Polymorphisms in the TNF-α receptor genes can influence anti-TNF-α treatment response among patients affected with IBD. However, some data regarding the impact of certain polymorphisms in these genes are divergent. For example, in a study where two cohorts from independent and prospective clinical trials were investigated different SNPs upon TNFRSF1A and TNFRSF1B were studied, and no significant association with the clinical response was found [47]. Moreover, other studies found that some TNF-α receptors polymorphisms could change the serum C-reactive protein (CRP) level, influencing the biological response. Specifically, in 344 CD patients and 152 UC patients, along with a group of 141 healthy volunteers, the prevalence of TNFR1 A36G and TNFR2 T587G polymorphisms were studied, since they were not studied in UC yet. However, even if the TNFR2 T587G allele was more frequently found in patients affected with UC, it was confirmed that these polymorphisms could not be considered as predictors of clinical response to the treatment, on the other hand, a lower biological response was seen in patients carrying the TNFR1 A36G polymorphism [48]. In a population of 121 Japanese patients in maintenance therapy with infliximab, the presence of the polymorphisms rs767455, rs976881, and rs1061622 was not statistically significant to prove their involvement in the response to infliximab maintenance therapy [44].

In a study on 104 subjects, 54 with CD and 50 healthy controls, the frequencies of some SNPs in TNFRS1A (rs4149584, rs767455, rs4149579) and 1B (rs1061622, rs1061624, rs3397) were analyzed. The results demonstrated a higher frequency of rs767455, rs1061624, and rs3397 polymorphisms in CD patients compared to controls (*p* < 0.05), with a downregulated expression of their corresponding genes, consistent with the downregulation of the receptors in CD patients [41].

The efficacy of anti-TNF-α inhibitors was also studied in 81 CD patients, where TNFRSF1A and TNFRSF1B polymorphisms rs767455, rs4149570, rs1061622, rs1061624, and rs3397 were analyzed. The presence of the G allele of the rs767455 polymorphism was associated with a reduced effect of infliximab, compared with the AA genotype (*p* < 0.01) [49]. In addition, the rs767455, rs1061622, rs1061624, and rs3397 polymorphisms were also analyzed in 297 CD patients from 7 centers around Spain. The frequency of the A allele in rs1061624 polymorphism was higher in non-responders (*p* = 0.02), while the CC genotype in the rs3397 was significantly higher in responders [50].

Furthermore, an observational cohort study conducted on 124 Caucasian CD patients under infliximab maintenance therapy showed that presence of the minor TNFRSF1B rs976881 allele was a negative predictor of efficacy (*p* = 0.014), especially in homozygosity (*p* = 0.006). Differently, the rs1061622 polymorphism positively influenced the response to infliximab (*p* = 0.014), also during maintenance therapy (*p* = 0.007) [51].

In summary, treatments are affected mainly by the presence of the A allele in -308 TNF-α disregarding of the anti-TNF used. Moreover, the presence of the G allele on TNFRSF1A was also associated with a poor response of treated patients, suggesting that a pre-evaluation could be useful for prescribing the most appropriate drug, according to the patient’s genetic profile (Table 1).

### 3.3. Polymorphisms on Innate Immunity Related Genes

Another gene identified as a susceptibility gene for the development of IBDs is the NOD2/CARD15 gene [23,52]. Three main variants (rs2066844, rs2066845, rs41450053) that are known to act as major genetic risk factor for CD have been investigated in relation to the efficacy of anti-TNF-α agents. In a cohort study with infliximab-treated 245 CD patients, with 45 non-responders, the 3 main variants in NOD2/CARD15 resulted as not predictive of response to infliximab [53]. Another study, on 24 patients treated with adalimumab, from a cohort of 165 CD patients, found no significative association between the same main variants in the NOD2/CARD15 gene and response to adalimumab [54]. These results were confirmed by other prospective clinical trials, for instance Mascheretti et al. included a total of 534 patients from two multicenter clinical trials to investigate an association between NOD2/CARD15 variants in response to infliximab. However, the NOD2/CARD15 genotype distribution was not different between responders and non-responders, therefore its role as predictor for clinical efficacy of infliximab has been excluded [55]. Moreover, a meta-analysis was performed on a total of 355 patients treated with either infliximab and/or adalimumab, confirming that NOD2/CARD15 mutations were not associated with response to TNF-inhibitors. Ultimately, it is thought that polymorphisms in this gene are not predictive of non-responsiveness to anti-TNF-α therapy [56].

Also TLR-4 and CD14 genes have been investigated for their possible involvement in response to anti-TNF therapies. To investigate the possible role of TLR-4 and CD14 polymorphism in the response to adalimumab, 24 patients, from a cohort of 165 CD subjects, were studied. TLR4 896 A/G and CD14 -260 C/T SNPs were screened, without, however, finding any relationship between their occurrence and the impact of treatment response [54]. Same conclusions were drawn by Walczak et al., who studied 107 CD Polish patients treated with infliximab and adalimumab [57]. However, more recently, in 587 CD and 458 UC Danish patients, TLR4 rs5030728 and rs1554973 polymorphisms were considered as predictors for the response to therapy [58].

A target molecule in anti-TNF therapy is IL-1β, thus the presence of the rs1143634 polymorphism was assayed in 47 patients with either CD or UC. The results showed that this mutation was related to higher serum IL-1β levels, possibly correlated with a decreased response to infliximab therapy [45]. Likewise, a polymorphism upon the gene encoding IL-1-receptor antagonist (IL-1RA), rs4251961, was associated with a poor response in patients with CD and UC (*p* = 0.049) [58]. On the other hand, the polymorphism rs4848306, resulting in reduced IL-1β circulating levels, enhanced the beneficial response to infliximab in IBD patients [59].

Reduced IL-6 expression due to the polymorphism rs10499563 was borderline associated (*p* = 0.05) with beneficial response in the combined study of 482 CD and 256 UC Danish patients [58].

As for other cytokines that may be affecting the response to these therapies, some studies have investigated the role of IL-11, IL-13, IL-17 and IL-27. In 103 Japanese patients, five SNPs in IL17A, eight in IL17RA and two in IL17RC were genotyped. A G/G genotype of rs766748 polymorphism was associated with a beneficial response after a year of treatment. However, none of the other polymorphisms was associated with clinical response to infliximab [60].

In summary, the G allele in rs5030728, the T allele in rs1554973, the C allele in rs10499563 and the A allele in rs4848306 were associated with a better response to infliximab. On the other hand, the C allele in rs1143634 was associated with a poor response to infliximab, suggesting that a pharmacogenetic pre-evaluation of patients could be useful for a targeted treatment of IBD patients (Table 2).

### 3.4. Polymorphisms on Apoptosis and Autophagy Genes

Apoptosis and autophagy genes, such as FASL, CASP9 and ATG16L1, have been studied to evaluate primary responses to anti-TNF-α therapy [61]. In a cohort of 287 CD patients treated with infliximab, the FasL gene polymorphisms in position -843 and the caspase-9 in position 93 have been associated with a positive response to infliximab treatment. Especially, the FasL -843 C/C or C/T genotype have been associated with a better response compared to T/T genotype (*p* = 0.002) [62]. Moreover, in the same study, patients carrying the caspase-9 93 T/T genotype showed a positive response to the therapy (*p* = 0.04), compared to C/C or C/T genotype [62]. A polymorphism in the ATG16L1 gene, rs10210302, was analyzed in 102 Slovenian CD patients administered with adalimumab. After 12 weeks of treatment, patients with C/T and T/T genotype showed a biological response to adalimumab, while the patients carrying the C/C genotype were not responders (*p* = 0.0008). Moreover, the presence of rs10210302, was associated with a better response to adalimumab even after 20 (*p* = 0.004) and 30 weeks (*p* = 0.04) [63]. Furthermore, in a study conducted on 94 pediatric CD and UC patients, the presence of the T allele in rs2241880 was associated with a poor response to infliximab and adalimumab treatment [64]. In summary, the presence of the C allele in rs763110 and in rs10210302 was associated with a better response to infliximab and adalimumab. On the other hand, the T allele in rs2241880 was associated with a poor response in IBD patients treated with infliximab and adalimumab suggesting that a pharmacogenetics pre-evaluation could be useful to improve the efficacy of these biological treatments in IBD patients (Table 3).

### 3.5. Pharmacogenetics of Anti-IL-12 and Anti-IL-23 Agents

Ustekinumab is an agent used in patients with moderate-to-severe CD that has been shown to have a significant clinical response. To the best of our knowledge, we found only one study investigating the role of SNPs upon the Protein Tyrosine Phosphatase Non-Receptor Type 2 (PTPN2) gene which could interfere with the response to anti-IL-12 and IL-23 therapy. The presence of SNPs on its locus is associated with chronic inflammatory conditions [65], therefore the role of rs2542151and rs7234029 polymorphisms was studied in an uncontrolled monocentric retrospective observational study including 379 patients with moderate-to-severe CD. An association between non-responders to this treatment and the presence of rs7234029 polymorphism was found.

In summary, the role of this polymorphism should be further investigated as a potential biomarker for response to ustekinumab.

### 3.6. Pharmacogenetics of Anti-Integrin Agents

Vedolizumab is a humanized immunoglobulin G1 monoclonal antibody to α_4_β_7_ integrin which modulates lymphocyte trafficking and therefore should decrease the inflammatory response in IBD patients. To date, there are no studies examining the associations between SNPs and treatment outcome of IBD patients with this biological drug.

## 4. Discussion and Future Perspectives

IBD comprises a heterogeneous group of subtypes with different molecular characteristics. Several genetic biomarkers were associated with anti-TNF-α treatment response in IBD and, generally, interpretation of genetic information in a meaningful way may be difficult [66]. The value of different genomic biomarkers has been evaluated, particularly those which may impact anti-TNF-α drug response, finding that not only the functional polymorphisms of TNF-α and TNFR could play a key role to the response following medical treatment, but also polymorphisms in cytokine and immune pathways. Despite they cannot be completely considered predictive biomarkers since they need to be validated in larger cohort of patients, genetic biomarkers generally hold the advantage of not changing over time and some of them seem very promising for future clinical practice.

Therefore, association between SNPs and response to biological treatment in IBDs was investigated by numerous pharmacogenetics studies, finding a linkage between some SNPs and response to biological therapy. In this systematic review, genetic polymorphisms associated with treatment outcome in IBD patients undergoing biological therapy, have been examined to suggest potential pharmacogenetics approach for predictive benefits. To date, there is no recommendation regarding the search for polymorphisms of genes involved in the pathogenesis of IBD as part of therapeutic optimization.

Our methodological evaluation suggests the use in clinical practice of some polymorphisms of interest in IBD patients. Particularly, polymorphisms on TNF-α and TNFR genes, such as the rs1800629, were associated with a poor response, as well as the rs1799724, rs767455, rs1061624 and rs976881. Contrarily, other polymorphisms in these genes have been associated with a better response, such as rs361525 and rs3397.

Most of the polymorphisms on innate immunity genes did not show any correlation with the clinical response; however, TLR4 rs5030728, rs1554973, IL-1β rs4848306 and IL-17 rs766748 (GG genotype) polymorphisms were associated with a better response, while the IL-1β rs1143634 polymorphism was related to a poor response.

Moreover, among autophagy and apoptosis related genes, we found that FasL rs763110, Caspase-9 rs4645983 and ATG16L1 rs10210302 polymorphisms were associated with a better response; on the other hand, ATG16L1 rs2241880 polymorphism was correlated to a poor response. Finally, the role of polymorphisms interfering with the activity of biologicals targeting IL-12 and IL-23 was also assessed despite only one study associated the PTPN2 rs7234029 polymorphism with Ustekinumab non-responders.

However, in this systematic review some limitations and biases are present: for instance, environmental factors such as nutrition, lifestyle, and other medication that may interact with genetic susceptibility were not considered. Moreover, the monitorization time, differences between populations, genetic heterogeneity and gene-gene interactions were not taken into account. Likewise, potential statistical errors in the analyzed studies may affect the results. Considering that the included studies were heterogenous for some characteristics (ethnicity, biological used, type of IBD), a possible bias can be ascribed. Moreover, some inevitable publication bias might exist in the results because only published studies were retrieved; as a matter of fact, preprint servers, other registries/results database (such as ClinicalTrials.gov and the International Clinical Trials Registry Platform) were not used and the search was limited to studies published in academic journals. We tried to exclude potentially overlapping data, however we may have missed some overlapping data or unintentionally excluded non-overlapping data. Moreover, the severity of disease activity in patients included in studies investigated may have differed between the studies, therefore this may have introduced other biases that have not been accounted for. Furthermore, we cannot exclude that associations were not found because of low statistical power in some studies included in our investigation.

However, this systematic review also carries strengths: we evaluated numerous polymorphisms which might modify the efficacy of all biological drugs approved for the management of IBD, providing a broad pharmacogenetic overview of current biological treatment for IBD. Moreover, all the candidate genes that were included in our investigation, having a known biological effect, allowed a logical interpretation of the observed effects. As a matter of fact, our study highlights associations between treatment responses and specific alleles based on a strong biological or clinical effect.

In conclusion, from the clinical perspective, improving TNF-α, TNFR and IL-1 pharmacogenetics would be the most suitable way to move towards a targeted therapy for IBDs, even if bias such as ethnicity and different kinds of biological agents used should be considered. Pre-treatment patients genotyping should be incorporated into clinical IBD management guidelines, as it is the most appropriate strategy to select the most suitable biological drug for an individual patient. Finally, clinical implementation of pre-treatment genetic tests could be achieved by investigating the role of target genes which could interfere with the action of other biologicals apart from TNF-α inhibitors, in order to identify more predictive genetic variants.

## Figures and Tables

**Table 1:** Summary of studies on pharmacogenetics of anti-TNF treatment in IBD, focusing on the TNF-α and TNFR genes.

Study	Number of Patients	Polymorphic Locus	Biological Agent	Clinical Effects
Netz et al., 2017 [40]	121	TNF-α rs1800629	Infliximab	The A allele in rs1800629 was associated with a poor response
López-Hernández et al., 2014 [41]	82	TNF-α rs1800629	Infliximab	The A allele in rs1800629 was associated with a poor response
Balog et al., 2004 [39]	14	TNF-α rs1800629	Infliximab	The A allele in rs1800629 was associated with a poor response
Song et al., 2015 [43]	476	TNF-α rs1800629 TNF-α rs361525 TNF-α rs1799724	Infliximab, Adalimumab	The G allele in rs1800629 and in rs361525 and the C allele in rs1799724 were associated with a better response
Matsuoka et al., 2018 [44]	121	TNF-α rs1799724	Infliximab	The T allele in rs1799724 was associated with a poor response
Pierik et al., 2004 [48]	637	TNFRSF1A rs767455	Infliximab	The G allele in rs767455 was associated with a poor response
Matsukura et al., 2008 [49]	81	TNFRSF1A rs767455	Infliximab	The G allele in rs767455 was associated with a poor response
Medrano et al., 2014 [50]	297	TNFRSF1B rs1061624 TNFRSF1B rs3397	Infliximab	The A allele in rs1061624 is associated with non-response, while the CC genotype in rs3397 is associated with a better response
Steenholdt et al., 2012 [51]	124	TNFRSF1B rs1061624 TNFRSF1B rs976881	Infliximab	The G allele in the rs1061624 is associated with a better response, while C allele in rs976881 is associated with a poor response

**Table 2:** Summary of studies on pharmacogenetics of anti-TNF treatment in IBD, focusing on the innate immunity-related genes.

Study	Number of Patients	Polymorphic Locus	Biological Agent	Clinical Effects
Bank et al. 2019 [58]	1045	TLR4 rs5030728 TLR4 rs1554973 IL-6 rs10499563 IL-1RA rs4251961	Infliximab	The G allele in rs5030728, the T allele in rs1554973 and the C allele in rs10499563 were associated with a better response, while the C allele in rs4251961 was associated with a poor response
Lacruz-Guzmán et al., 2013 [45]	47	IL-1β rs1143634	Infliximab	The C allele in rs1143634 was associated with a poor response
Bank et al., 2014 [59]	738	IL-1β rs4848306	Infliximab	The A allele in rs4848306 was associated with a better response
Urabe et al., 2015 [60]	103	IL-17 rs766748	Infliximab	The G/G genotype in rs766748 was associated with a better response

**Table 3:** Summary of studies on pharmacogenetics of anti-TNF treatment in IBD, focusing on the autophagy and apoptosis related genes.

Study	Number of Patients	Polymorphic Locus	Biological Agent	Clinical Effect
Hlavaty et al., 2005 [62]	287	FasL rs763110 Caspase-9 rs4645983	Infliximab	The C/C and C/T genotypes in rs763110 and the T/T genotype in rs4645983 were associated with a better response
Koder et al., 2015 [63]	102	ATG16L1 rs10210302	Adalimumab	The C/T and T/T genotypes in rs10210302 were associated with a better response
Dubinsky et al., 2010 [64]	94	ATG16L1 rs2241880	Infliximab, Adalimumab	The C/T and T/T genotypes in rs2241880 were associated with a poor response
